# The Use of Lactic Acid Bacteria Starter Culture in the Production of *Nunu*, a Spontaneously Fermented Milk Product in Ghana

**DOI:** 10.1155/2014/721067

**Published:** 2014-12-02

**Authors:** Fortune Akabanda, James Owusu-Kwarteng, Kwaku Tano-Debrah, Charles Parkouda, Lene Jespersen

**Affiliations:** ^1^Department of Nutrition and Food Science, University of Ghana, P.O. Box LG 25, Legon, Ghana; ^2^Department of Applied Biology, Faculty of Applied Sciences, University for Development Studies, Navrongo Campus, P.O. Box 24, Navrongo, Ghana; ^3^Food Technology Department (DTA/IRSAT/CNRST), BP 7074, Ouagadougou 03, Burkina Faso; ^4^Department of Food Science, Food Microbiology, Faculty of Science, University of Copenhagen, Rolighedsvej 30, 1958 Frederiksberg C, Denmark

## Abstract

*Nunu*, a spontaneously fermented yoghurt-like product, is produced and consumed in parts of West Africa. A total of 373 predominant lactic acid bacteria (LAB) previously isolated and identified from *Nunu* product were assessed *in vitro* for their technological properties (acidification, exopolysaccharides production, lipolysis, proteolysis and antimicrobial activities). Following the determination of technological properties, *Lactobacillus fermentum* 22-16, *Lactobacillus plantarum* 8-2, *Lactobacillus helveticus* 22-7, and *Leuconostoc mesenteroides* 14-11 were used as single and combined starter cultures for *Nunu* fermentation. Starter culture fermented *Nunu* samples were assessed for amino acids profile and rate of acidification and were subsequently evaluated for consumer acceptability. For acidification properties, 82%, 59%, 34%, and 20% of strains belonging to *Lactobacillus helveticus, L. plantarum, L. fermentum*, and *Leu. mesenteriodes*, respectively, demonstrated fast acidification properties. High proteolytic activity (>100 to 150 *μ*g/mL) was observed for 50% *Leu. mesenteroides,* 40% *L. fermentum,* 41% *L. helveticus*, 27% *L. plantarum,* and 10% *Ent. faecium* species. In starter culture fermented *Nunu* samples, all amino acids determined were detected in *Nunu* fermented with single starters of *L. plantarum* and *L. helveticus* and combined starter of *L. fermntum* and *L. helveticus*. Consumer sensory analysis showed varying degrees of acceptability for *Nunu* fermented with the different starter cultures.

## 1. Introduction

*Nunu* is a spontaneously fermented milk (yoghurt-like) product in Ghana and other parts of West Africa including Nigeria and Burkina Faso. Unlike other African fermented milk products where milk of goats, sheep, and camels is used,* Nunu* is solely prepared from cow milk. The traditional processing of* Nunu* involves collecting fresh cow milk into containers and then allowing it to ferment for a day or two days at ambient temperature.* Nunu* is yoghurt-like in taste (a sharp acid taste) and it can be taken alone or with* Fura* [1, 2]. Like many other spontaneously fermented foods in Africa, the production of* Nunu* is largely home-based and the fermentation is spontaneous. Thus, starter cultures are not available, but old stocks of previous ferments and fermentation containers are used to initiate fermentation in new batches. The dependence on such undefined and diverse microbial consortium during* Nunu* fermentation may result in product of variable quality and stability.

Currently, there is no information on the use of starter cultures for* Nunu* fermentation. However, few investigations have been carried out on the microbiology of Ghanaian traditionally fermented milk products [2–4]. The predominant microorganisms isolated from this traditionally fermented milk should be developed into starter cultures that could be used to produce fermented milk products of consistent quality and consumer acceptability. Thus, it should be possible to improve the quality and consumer acceptability of* Nunu* through controlled fermentation using starter culture. The culture should, however, be well-defined. Such starter cultures must be developed with a clear understanding of the ecology of the microbial species associated with the desirable traditional fermentation process, and their contributions to the products safety and quality are determined. The first stage in designing such starter culture(s) is to characterize and identify the technologically important microorganisms associated with the traditional fermentation of the product and then to test the use of the identified organisms in fermentation trials.

The objective of the present study was therefore to evaluate the technological potential of lactic acid bacteria isolated from spontaneously fermented* Nunu* in view of their application as starter cultures in* Nunu* production.

## 2. Materials and Methods

### 2.1. Determination of Technological Properties of LAB

A total of 373 LAB isolated and identified from spontaneously fermented* Nunu* in Ghana using a combination of phenotypic and genotypic characteristics [2] were assessed for their technological properties. The LAB species included 174* L. fermentum*, 44* L. plantarum*, 40* Ent. faecium*, 41* L. mesenteroides*, 35* L. helveticus*, and 39* Ent. italicus*.

#### 2.1.1. Acidification Properties

Acidification properties of the LAB were measured by the change in pH with time [5]. The strains were initially grown in MRS broth and then in sterile reconstituted skim milk supplemented with yeast extract (0.3%) and glucose (0.2%) for two successive subcultures. Sterile reconstituted skim milk (100 mL) was inoculated with 1% of a 24 h activated culture and pH changes were determined using pH meters (Crison Basic, Barcelona) during incubation at 30°C. Measurement of pH was carried out in triplicate at 2 h intervals for 24 h. The acidification rate was calculated as ΔpH; ΔpH = pH_at time_ − pH_zero time_. The cultures were considered as fast, medium, or slow acidifying when a ΔpH of 0.4 U was achieved at 3, 3–5, and >5 h, respectively.

#### 2.1.2. Proteolytic Activity

The proteolytic activity of the isolates during fermentation of milk was measured by assessing the free amino groups using the o-phthaldialdehyde (OPA) method [6]. Briefly, 3 mL aliquots of the samples were mixed with 3 mL of 1% (w/v) TCA (trichloroacetic acid) and filtered using a filter paper (Dublin, CA, USA). The filtrate was collected and approximately 150 *µ*L was added to 3 mL of OPA reagent. The mixture was held at room temperature (~20°C) for 2 minutes and the absorbance of each solution was measured by using a spectrophotometer (Melbourne, Australia) at 340 nm. The proteolytic activity of the bacterial cultures was expressed as the absorbance of OPA derivatives at 340 nm. A relative degree of proteolysis was determined as the difference between proteolytic activities in fermented milk to that of untreated milk.

#### 2.1.3. Lipolytic Activity

Strains were grown overnight at 37°C in MRS broth. A loopful of fresh culture was placed on tributyrin agar [7]. Plates were incubated at 37°C for 4 days and observed daily for halo formation around the colonies. The radius of the halo formation (in mm) at the end of incubation was measured.

#### 2.1.4. Antimicrobial Activities of LAB

*(1) Indicator Strains*. The indicator strains included* Bacillus cereus* PA24,* Staphylococcus aureus* ATCC 19095,* Escherichia coli* O157:H7,* Listeria monocytogenes* Scott A,* Salmonella typhi* ATCC 13311, and* Pseudomonas aeruginosa* BFE 162.* Bacillus cereus* PA24,* Escherichia coli* O157:H7, and* Pseudomonas aeruginosa* BFE 162 were obtained from the Department of Applied Biology, DANIDA Microbiology Laboratory of the University for Development Studies, while* Staphylococcus aureus* ATCC 19095,* Listeria monocytogenes* Scott A, and* Salmonella typhi* ATCC 13311 were obtained from the Department of Nutrition and Food Science of the University of Ghana.

*(2) Preparation of Cell-Free Supernatant (CFS)*. Each LAB isolate was inoculated in 10 mL of MRS broth and incubated at 30°C for 48 hrs. After incubation, a cell-free supernatant was obtained by centrifuging the bacterial culture at 6000 ×g for 15 min followed by filtration of the supernatant through 0.20 *µ*m pore size syringe filters (Sartorius, Minisart, Göttingen, Germany).

*(3) Screening for Antimicrobial Activities*. The agar-well diffusion method was employed in the screening of LAB for antimicrobial activities. Indicator lawns were prepared by inoculating 20 mL of BHI molten agar media with 100 *μ*L (approximately 10^7^ cfu/mL) of an overnight culture of each indicator organism and allowing them to solidify in a Petri dish. Wells were cut into the agar with a sterile 6 mm diameter cork-borer and sealed with two drops of sterile agar. Fifty microliters (50 *μ*L) of the filtered cell-free supernatant of test strains was separately placed into the wells. The plates, prepared in duplicate, were kept at 4°C for 24 h [8] to allow prediffusion of the CFS into the agar and then incubated at 37°C for 24 h. They were then observed for possible clearing of zones (inhibition zones). The antimicrobial activity was determined by measuring the diameter of the inhibition zones around the well using caliper in mm. Results were recorded as no inhibition (−), weak inhibition (+), moderate inhibition (++), and strong inhibition (+++) when the diameter is <1–4 mm, >4–8 mm, and >8–12 mm, respectively.

#### 2.1.5. Isolation, Purification, and Quantification of EPS Produced by the LAB

The screening of the isolates for EPSs production was carried out according to the method described by Guiraud [9]. The isolates cultured on MRS agar were streaked onto LTV agar (0.5% (w/v) tryptone (Merck), 1% (w/v) meat extract (Merck), 0.65% (w/v) NaCl (Merck), 0.8% (w/v) potassium nitrate (Merck), 0.8% (w/v) sucrose (Merck), 0.1% (v/v) Tween 80 (Merck), and 1.7% (w/v) agar (Merck), pH 7.1 ± 0.2) and incubated at 30°C for 48 h. The sticky aspect of the colonies was determined by testing them for slime formation using the inoculated loop method [10]. The isolates were considered positively slimy producer if the length of slime was above 1.5 mm.

The positive isolates were confirmed growing them on MRS sucrose broth and incubating them at 30°C for 24 h. A volume of 1.5 mL of the 24 h culture was centrifuged at 5000 g for 10 min (4°C) and 1 mL of the supernatant was put in a glass tube and an equal volume of ethanol 95% was added. An opaque link formed at the interface of the tube indicates the presence of EPSs. Samples were centrifuged 2500 ×g for 20 min and the pellets were dried at 100°C. The total carbohydrate content of the EPS was determined using the phenol-sulfuric acid procedure of Dubois et al. [11]. Briefly, two milliliters of sample solution was pipetted into a colorimetric tube, and 0.05 mL of 80% phenol was added. Then 5 mL of concentrated sulfuric acid was added rapidly, with the stream of acid directed against the liquid surface rather than against the side of the test tube in order to obtain good mixing. The tubes were then allowed to stand for 10 minutes, and then they were shaken and placed for 10 to 20 minutes in a water bath at 25° to 30°C before readings were taken. The absorbance of the characteristic yellow orange color was measured at 490 nm. Distilled water was used as control. The amount of EPS was expressed in *μ*g/mL.

### 2.2. Starter Culture Fermentation of Milk with Selected LAB 

#### 2.2.1. Selection of LAB Strains

Four (4) strains of LAB were selected and used as starter cultures during the fermentation of milk to produce* Nunu*. These include* Lactobacillus fermentum* 22-16 (LF-22-16),* Lactobacillus plantarum* 8-2 (LP-8-2),* Lactobacillus helveticus* 22-7 (LH-22-7), and* Leuconostoc mesenteroides* 14-11 (LM-14-11). The strains were selected based on their predominance in traditional spontaneous* Nunu* fermentation as well as their desirable technological properties including faster rates of acidification, high proteolytic and low lipolytic activities, ability to produce exopolysaccharides, and the possession of antimicrobial properties in milk.

#### 2.2.2. Preparation of Starter Cultures and Fermentation of Milk

The LAB strains to be used as inocula were prepared by transferring a loopful of an overnight culture from MRS agar into 10 mL MRS broth and incubated at 35°C for 24 h. One hundred microliters of the 24 h old culture was transferred into 10 mL MRS broth and incubated at 35°C for 16 h (overnight). Subsequently, cells were harvested by centrifugation at 5000 g for 10 min (4°C); washed three times with 20 mL sterile diluent (Merck), pH 7.2 ± 0.2; and finally suspended in 10 mL of sterile diluent, and these served as the isolate inocula. Flasks containing 500 mL of fresh milk pasteurized at 60°C for 30 min in a water bath were inoculated in duplicate at 35°C for 24 h. Single and combined starter cultures used for the fermentation of milk to produce* Nunu* are shown in Table 1.

#### 2.2.3. Rate of Acidification during* Nunu* Fermentation with Starter Cultures

The pH of fermenting* Nunu* was determined using a digital pH meter (Crison basic 20, Barcelona). The pH meter was calibrated using standard buffer solutions and measurements were taken at ambient temperature of 30 ± 2°C. All measurements were carried out in triplicate and means and standard deviations were determined.

#### 2.2.4. Determination of Amino Acids Profile

*(1) Preparation of Samples.* Ten milliliters of* Nunu* samples was defatted with distilled petroleum ether (Labscan, Dublin, Ireland) in a Soxhlet apparatus and stored in screw-capped plastic tubes at −20°C until they were required. Preparation of each sample was carried out in triplicate.

*(2) Analysis of Free-Amino Acids Profile*. The free-amino acids profile analysis of the samples was assayed according to Ojinnaka and Ojimelukwe, [12] with minor modifications. First of all, the samples were hydrolyzed by weighing 0.5 g into 20 mL volumetric flask. The volumetric flask was then filled to the mark with 0.1 M hydrochloric acid and was then shaken thoroughly to mix. The content was left over night to extract; 1 mL of the overnight sample was filtered through a 0.45 *μ*m filter. Ten microliters of the filtrate was then placed in a sample vial for drying and redrying. The hydrolyzed dried samples were derivatized automatically on the Waters HPLC by allowing the samples to react, under basic situations with phenylisothiocyanate (i.e., PITC) to get phenylthiocarbamide (PTC) amino acid derivatives. The duration for this reaction was 45 minutes per sample, as calibrated on the instrument. A set of standard solutions of the amino acids were prepared from Pierce Reference standards H (5 *μ*L) into autosampler cups and they were also derivatized. These standards (200 *μ*L, 250 *μ*L, 300 *μ*L, and 400 *μ*L) were used to generate a calibration file that was used to determine the amino acid contents of the samples. After the derivatization, a methanol solution (1.5 N), containing the PTC amino acids, was transferred to a narrow bore (Waters 600) HPLC system for separation. The separation and identification of amino acids were done in reverse phase C18 silica column and the analytes were detected at the wavelength of 254 nm. The elution of the whole amino acids in the samples took 12 minutes. The buffer system used for separation was 140 mM sodium acetate pH 6.40 as buffer A and 80% acetonitrile as buffer B. The program was run using a gradient of buffer A and buffer B concentration and ending with a 55% buffer B concentration at the end of the gradient. The intensity of the chromatographic peaks areas were automatically and digitally identified and quantified using Empowers 2 software data analysis system which was attached to the Waters 600 HPLC System. The calibration curve or file prepared from the average values of the retention times (in minutes) and areas (in Au) of the amino acids in 5 standards runs was used. Since a known amount of each amino acid in the standard was loaded into the HPLC, a response factor (Au/pmol) was calculated by Empowers 2 software that was interphased with the HPLC. This response factor was used to calculate the amount of each of the amino acid (in pmol) in the sample. The amount of each amino acid in the sample was finally calculated by the software by dividing the intensity of the peak area of each by the internal standard in the chromatogram and multiplying this by the total amount of internal standard added to the original sample.

#### 2.2.5. Consumer Sensory Evaluation of* Nunu*

*Nunu* products prepared by fermentation with different starter cultures were served to 35 volunteered untrained panelists (drawn from the Faculty of Applied Sciences of the University for Development Studies and the Navrongo Community) who are familiar with* Nunu*. The panel independently, in separate sensory evaluation booths, evaluated the various products for their sensory qualities including taste, colour/appearance, odour, texture, and overall acceptability, using a nine-point hedonic scale (1, 5, and 9 represent dislike extremely, neither like nor dislike, and like extremely, resp.). All eleven* Nunu* products were presented to the panelists randomly placed side-by-side, with each panelist receiving 2 rounds of each product and water for rinsing. Spontaneously fermented* Nunu* (without added known starter culture) served as control sample. Before tasting the products, panelists were asked to evaluate the products' appearance using a 9-point hedonic scale ranging from “dislike extremely” to “like extremely.” After judging appearance, the panelists were then allowed to taste the samples and evaluate other sensory properties using a 9-point hedonic scale, once again ranging from “dislike extremely” to “like extremely.” The judges were made to wash their mouth with water after evaluating each product.

### 2.3. Statistical Analysis

All analyses were carried out in triplicate. Data obtained were subjected to one-way analysis of variance (ANOVA) and means were separated by Tukey's family error rate multiple comparison test (*P* < 0.05) using the MINITAB statistical software package (MINITAB Inc. Release 14 for windows, 2004).

## 3. Results and Discussion

### 3.1. Technological Properties of LAB Isolated from* Nunu*

#### 3.1.1. Acidification Properties

**Figure 1:**
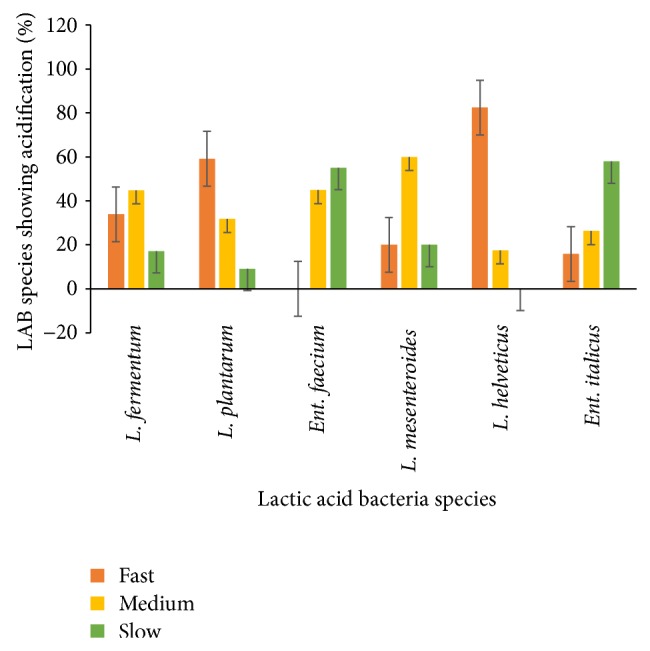
Acidification properties of predominant LAB species isolated from* Nunu*: fast, medium, and slow, when a pH of 0.4 U was achieved after 3 h, 6 h, and 24 h, respectively.

Rapid acidification is a priority for development of starter cultures for fermented milk products. The acidification properties of predominant LAB isolated from spontaneously fermented* Nunu* are shown in Figure 1. Generally, the rate of acidification varied among the isolates tested. Eighty-two percent (82%) of* Lactobacillus helveticus* were acidifying fast. Additionally, 34% of* L. fermentum*, 59% of* L. plantarum*, and 20% of* L. mesenteroides* demonstrated fast acidification properties as well. Idoui and Karam [13] indicated that* L*.* plantarum* and* L*.* curvatus* isolated from Jijel's traditional butter made from cows' milk were the fastest acid producers. In a study by Haddadin [14],* L*.* plantarum* was the fastest acid producing isolated strain. Sixteen percent (16%) of* Ent. italicus* were fast acidifiers while no strain of* Ent. faecium* showed fast acidification. These results are in agreement with Sarantinopoulos et al. [15], who indicated that* Enterococcus* strains were poor acidifiers in milk and* Ent. faecium* and* Enterococcus faecalis* have been reported to degrade lactose in milk slowly [16]. From the results obtained, a majority of strains showed fast rate of acidification as they were able to produce a ΔpH of 0.4 U after 3 h. A rapid decrease in pH is essential for coagulation and prevention or reduction of growth of adventitious microflora in yoghurt production. The fast acidifying strains are therefore good candidates for dairy fermentation process as primary starter culture, while poor acidification strains can be used as adjunct cultures depending on other properties [5]. Generally, the desirable characteristics for industrial LAB or starter are the abilities to rapidly and completely convert the raw materials into lactic acid with minimal nutritional requirements. A rapid acidification of the raw material prevents growth of undesirable microorganisms and is also essential for aroma, texture, and flavor of the end-product (*Nunu*). It has been observed that the faster the decrease in pH to <4, the faster the growth inhibition of the fermenting medium against pathogens such as* Salmonella spp*. [17].

#### 3.1.2. Proteolytic Activities

**Figure 2:**
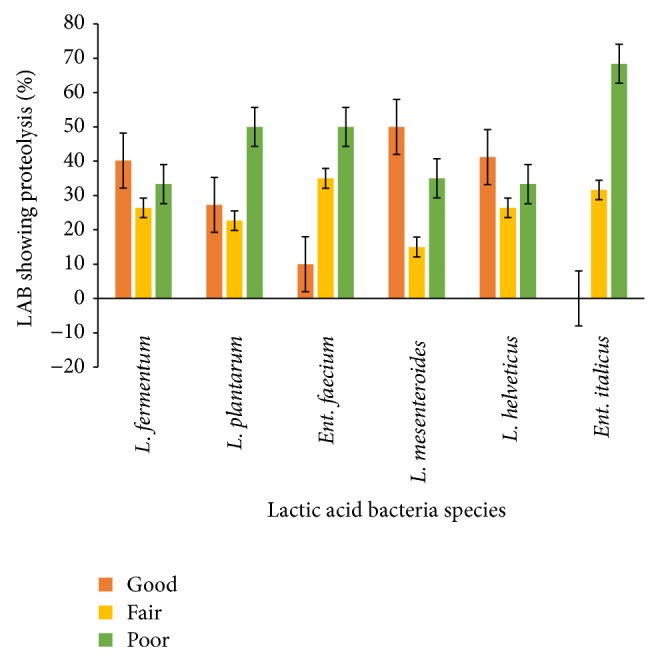
Pattern of proteolytic activities of predominant LAB isolated from* Nunu*. Good => 100–150 *μ*g tyrosine/mL, fair => 50–100 *μ*g tyrosine/mL, and Poor = 0–50 *μ*g tyrosine/mL.

In general most of the strains tested showed proteolytic activities to varying degrees (Figure 2). Forty percent (40%) of* L. fermentum*, 27% of* L. plantarum*, 10% of* Ent*.* faecium*, 50% of* L. mesenteroides*, and 41% of* L. helveticus* showed good (>100–150 *μ*g tyrosine/mL) proteolytic activities. The result is in contrast with those of Durlu-Ozkaya et al. [18] and Dagdemir and Ozdemir [19] who reported high proteolytic activity for LAB isolated from cheese. The contrast in proteolytic activities may be due to the strains associated with the products. Peterson et al. [20] reported that important differences exist between species of LAB in terms of the types and quantities of peptidase activities. The proteolytic activity of dairy lactic acid bacteria is essential for the bacterial growth in milk and is involved in the development of organoleptic properties of different fermented milk products [21, 22]. The production of high quality fermented dairy products depends on proteolytic systems of starter bacteria, since peptidase and amino acids formed have a direct impact on flavor or serve as flavor precursors in these products.

#### 3.1.3. Lipolytic Activities

Lipolytic activities were recognized by the presence of a clear halo in the tributyrin agar plates. Out of the 174 strains of* L*.* fermentum* screened for lipolytic activities, 32% were negative (showed no clear zones on the agar plates). Similarly,* L. plantarum* and* L. helveticus* had 9% and 8.6% strains not showing lipolytic activities, respectively (Table 2). Generally, majority of the strains tested for lipolysis were positive.

Very few studies on lactic acid bacterial lipases have been carried out. Tsakalidou et al. [23] concluded that even though LAB are weakly lipolytic, the* enterococcal* strains showed a significantly higher activity than the strains of most other genera of LAB. Microbial lipases are used in the dairy industry extensively for the hydrolysis of milk fat, and current applications include acceleration of cheese ripening and lipolysis of butter, fat, and cream [24]. The ability of LAB to show lipolytic activity* in vitro* is very promising. It is assumed that such activity can be manifested by the isolates* in vivo* which will lead to the reduction of cholesterol level inhumans if used as a starter or an adjunct culture [25].

#### 3.1.4. Exopolysaccharide Production

**Figure 3:**
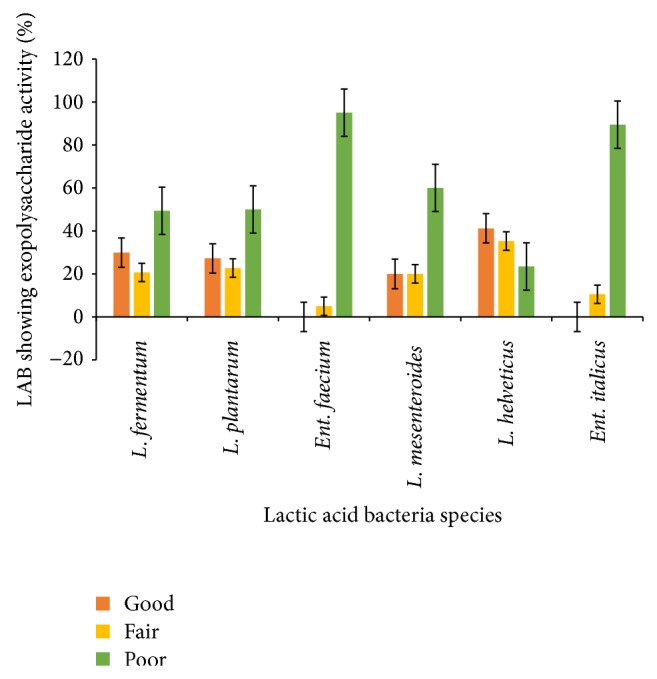
Exopolysaccharides activities of predominant LAB isolated from* Nunu*. Good => 100–150 *μ*g/mL, fair => 50–100 *μ*g/mL, and poor = 0–50 *μ*g/mL.

As shown in Figure 3, all the groups of LAB strains tested produced exopolysaccharide to some extent. Strains of* L. helveticus* (38%),* L. fermentum* (30%),* L. plantarum* (27%) and* L. mesenteroides* (18%) demonstrated good (>100–150 *μ*g/mL) exopolysaccharide production abilities. Most of the* Ent. faecium* (95%) and* Ent. italicus* (85%) strains were poor producers, producing below 50 *μ*g/mL of exopolysaccharides. Exopolysaccharide production is a desirable feature of bacteria applied in dairy products because EPSs act as natural biothickener leading to higher consistency and viscosity of the product and reduced syneresis [26]. However, most of them are chemically or enzymatically modified in order to improve their rheological properties (e.g., cellulose, starch, pectin, alginate, and carrageenan) and, therefore, their use is strongly restricted for food applications. An alternative source of biopolymers is microbial EPS. The EPSs of microbial origin have unique rheological properties because of their capability of forming very viscous solutions at low concentration and their pseudoplastic nature [27]. Some strains of LAB have been reported to produce EPS and gain increasing attention over the last few years because of their contribution to the rheology and texture of fermented milk and food products [28]. Most of the LAB producing EPS belong to the genera* Streptococcus*,* Lactobacillus*,* Lactococcus*,* Leuconostoc*, and* Pediococcus* [29]. EPS-producing LAB have a greater ability to withstand technological stresses [30] and survive the passage through the gastrointestinal tract compared to their non-producing bacteria. Additionally, EPS may induce positive physiological responses including lower cholesterol levels [31, 32], reduce formation of pathogenic biofilms [33] and modulation of adhesion to epithelial cells [34], and increase levels of* bifidobacteria* showing prebiotic potential [35, 36]. Hence, the choice of EPS-producing starter culture seems to give several advantages over nonproducing ones.

#### 3.1.5. Antimicrobial Activities

**Figure 4:**
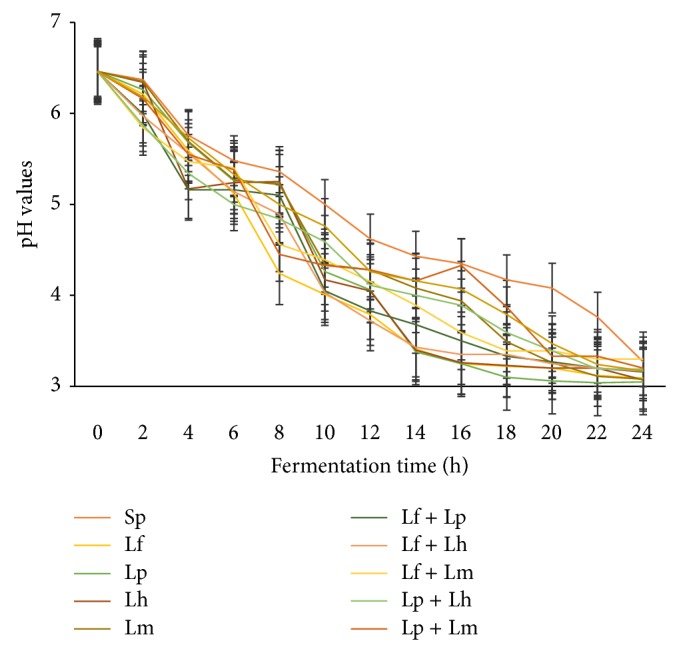
pH of fermenting milk with different starter cultures. Spontaneously fermented (Sp),* L. fermentum* (Lf),* L. plantarum* (Lp),* L. helveticus* (Lh), and* L. mesenteroides* (Lm),* L. fermentum* +* L. plantarum* (Lf + Lp),* L. fermentum* +* L. helveticus* (Lf + Lh),* L. fermentum* +* L. mesenteroides* (Lf + Lm),* L. plantarum* +* L. helveticus* (Lp + Lh),* L. plantarum* +* L. mesenteroides* (Lp + Lm),* L. helveticus*+* L. mesenteroides* (Lh + Lm).

The antimicrobial properties of predominant LAB isolated from* Nunu* are shown in Table 3. The LAB strains were able to inhibit the selected indicator organisms to varying degrees and Figure 4 illustrates the zones of inhibition. Similar to our findings, Kivanç [37] and Tadesse et al. [38] observed varying degrees of inhibition of various food borne pathogens by cell-free filtrates of LAB. Afolabi et al. [39] showed that antimicrobial producing microorganisms had the ability to inhibit the growth of other bacteria which included both Gram-negative and Gram-positive bacteria. Such antimicrobial activities were also demonstrated in the works of other researchers such as Adesokan et al. [40] where LAB species were tested against* Staphylococcus aureus*,* Pseudomonas aeruginosa*,* Candida albicans*,* Escherichia coli*, and* Proteus vulgaris*. Raccah et al. [41], Smith and Palumbo [42], and Cintas et al. [43] have demonstrated that the antimicrobial compounds produced by LAB can inhibit the growth of pathogenic bacteria of possible contaminants in fermented products. The ability to inhibit other organisms is due to the fact that LAB produces substances which are injurious to the indicator organisms depending on the concentration or quantity produced. These substances serve as competitive advantage to LAB when in mixed culture especially during fermentation and hence the dominance of LAB during fermentation of milk, cereals and vegetables. Wakil and Osamwonyi [44] indicated that LAB isolates showing antimicrobial activity were discovered to produce antimicrobial substances like lactic acid, hydrogen peroxide, and diacetyl, showing that the ability to inhibit other organisms was directly related to the ability of these organisms to produce these substances. Daeschel [45] reported the ability of LAB to produce lactic acid, thereby reducing the pH of the fermenting medium. The lactic acid produced serves to reduce the pH of the medium, thereby making it acidic which is not conducive for the survival of spoilage bacteria which may have found their way into the fermenting substrate during spontaneous fermentation. Lactic acid is a natural preservative that inhibits putrefying bacteria and is responsible for the improved microbiological stability and safety of the food. The acidity also leads to the souring of the final product which is characteristic of fermented products. Hydrogen peroxide produced adds to the antimicrobial activity of LAB and in some cases is a precursor for the production of other potent antimicrobial compounds such as super oxide (O2−) and hydroxyl (OH−) radicals [46, 47].

### 3.2. Impact of the Use of Starter Culture on Quality and Consumer Acceptability of* Nunu*

#### 3.2.1. Acidification of* Nunu*

The rates of acidification of* Nunu* prepared with different single and combined starter cultures are shown in Figure 4. Generally, there was a fast decrease in acidity with time of the milk using the starter cultures. The starter cultures were able to reduce the pH from approximately 6.46 to about 3.72 after 12 hours of fermentation, thus reducing the fermentation time. Lf, Lf + Lp, and Lf + Lh were the fast acidifiers.

The rate of acid development is a critical factor in milk fermentation. The rapid acidification of the raw material prevents growth of undesirable microorganisms and is also essential for aroma, texture, and flavor of the end-product.

#### 3.2.2. Amino Acid Profile

Table 4 summarizes the amino acid profile of* Nunu* prepared with various single and combined starter cultures. The results from this study show that the free amino acid profiles of* Nunu* produced with different starter cultures varied comparatively and were significantly different. All amino acids determined were detected in samples produced by fermenting milk with single starter cultures of* L. plantarum* (LP) and* L. helveticus* (LH) as well as the sample produced with a combined starter culture of* L. fermentum* and* L. helveticus* (LF + LH). Lysine, methionine, isoleucine, proline, glutamine, asparagine, alanine, and leucine were detected in all* Nunu* samples irrespective of the starter culture used. Except serine, all amino acids determined were detected in spontaneously fermented* Nunu* at varying concentrations. Many studies have shown that concentrations of most of the amino acids slightly increase due to fermentation. Muradyan et al. [48] reported that fermentation of milk by thermophilic lactic streptococci or acidophilic rods enriched the final products with at least 4 amino acids (cysteine, valine, proline, and arginine). An obligatory dietary requirement exists for tryptophan, leucine, isoleucine, valine, phenylalanine, methionine, lysine, threonine, and histidine. The last three of these indispensable groups of amino acids cannot be transaminated and so they must be supplied in the diet as such [49]. It was also observed that the samples contained the essential amino acids, namely, lysine, isoleucine, leucine, histidine, valine, threonine, methionine, and phenylalanine, while the nonessential amino acids detected were arginine, aspartic acid, serine, glutamic acid, proline, glycine, alanine, cysteine, and tyrosine. Milk is essentially rich in essential amino acids and branched chain amino acids. There is evidence that these amino acids have unique roles in human metabolism. In addition to providing substrates for protein synthesis, suppressing protein catabolism, and serving as substrates for gluconeogenesis, they also trigger muscle protein synthesis and promote protein synthesis [50]. The results suggest that* Nunu* produced with LAB starter culture can serve as a good source of essential and nonessential amino acids required in metabolism. The amino acids indicated as essential nutrients for infant growth [51], such as histidine, arginine, cysteine, and tryptophan, were also found in* Nunu*. Sulphur-containing amino acids (methionine and cysteine) are the most critical essential amino acids because they were easily lost from the body [52] and therefore their supplementation into food is greatly required. The results in this study however show that methionine was detected in all* Nunu* samples, but cysteine was not detected in* Nunu* fermented with* L. mesenteroides* (LM) starter culture and the combined starter culture of* L. fermentum* and* L. mesenteroides* (LF + LM).

#### 3.2.3. Consumer Sensory Evaluation of* Nunu*

Consumer sensory analysis showed varying degrees of acceptability for* Nunu* fermented with the different starter cultures in relation to their sensory attributes such as taste, odour, colour, texture, and overall acceptability (Table 5). Generally,* Nunu* fermented with lactic acid bacteria starter cultures, either single or combined, showed improved acceptability as compared to the spontaneously fermented* Nunu*. However,* Nunu* produced with single starter culture of* L. helveticus* or combined starter cultures of* L. fermentum* and* L. helveticus* (LF + LH) and* L. fermentum* and* L. plantarum* (LF + LP) showed significantly higher overall acceptability.

## 4. Conclusion

Lactic acid bacteria isolated from Ghanaian traditional fermented milk product (*Nunu*) have demonstrated desirable technological properties and have subsequently been successfully used as starter cultures for* Nunu* fermentation.* Lactobacillus fermentum*,* L. helveticus*, and* L. plantarum* starter cultures (whether used alone or in combination) were able to produce yoghurt with desirable consumer sensory characteristics. Therefore, further development and application of these cultures in commercial* Nunu *production will improve safety and consumer acceptability of the product.

## Figures and Tables

**Table 1:** Starter cultures used for *Nunu* fermentation.

Type of fermentation	Starter cultures	Codes
Single starters cultures	*Lactobacillus fermentum *	LF-22-16
	*Lactobacillus plantarum *	LP-8-2
	*Lactobacillus helveticus *	LH-22-7
	*Leuconostoc mesenteroides *	LM-14-11

Combined starter cultures	*Lactobacillus fermentum* + *Lactobacillus plantarum *	LF-22-16 + LP-8-2
	*Lactobacillus fermentum* + *Lactobacillus helveticus *	LF-22-16 + LH-22-7
	*Lactobacillus fermentum* + *Leuconostoc mesenteroides *	LF-22-16 + LM-14-11
	*Lactobacillus plantarum* + *Lactobacillus helveticus *	LP-8-2 + LH-22-7
	*Lactobacillus plantarum* + *Leuconostoc mesenteroides *	LP-22-7 + LM-14-11
	*Lactobacillus helveticus* + *Leuconostoc mesenteroides *	LH-22-7 + LM-14-11

No starter culture was added	Spontaneously fermented	SP

**Table 2:** Lipolytic activities of predominant LAB isolated from *Nunu. *

LAB species	Zones of inhibition		
	No inhibition	1–3 cm	>3–5 cm
*L. fermentum* (*n* = 174)	56	100	18
*L. plantarum* (*n* = 44)	4	28	12
*Ent. faecium* (*n* = 40)	9	24	7
*L. mesenteroides* (*n* = 41)	9	24	8
*L. helveticus* (*n* = 35)	3	21	11
*Ent. italicus* (*n* = 39)	7	30	2

*n*: number of organisms used.

**Table 3:** Antimicrobial activities of predominant LAB against selected pathogenic microorganisms.

Number of LAB tested	^*^Range of inhibition of pathogens					
	*B. cereus *	*E. coli *	*L. monocytogenes *	*S. typhi *	*Staph. aureus *	*P. aeruginosa *
*L. fermentum* (174)	8 (+)	4 (+)	6 (+)	2 (+)	6 (+)	4 (+)
	2 (++)	0 (++)	2 (++)	0 (++)	2 (++)	0 (++)
	0 (+++)	0 (+++)	0 (+++)	0 (+++)	0 (+++)	0 (+++)
	164 (ND)	170 (ND)	166 (ND)	172 (ND)	166 (ND)	170 (ND)

*L. plantarum* (44)	18 (+)	4 (+)	6 (+)	2 (+)	6 (+)	2 (+)
	2 (++)	2 (++)	4 (++)	2 (++)	2 (++)	2 (++)
	0 (+++)	0 (+++)	2 (+++)	0 (+++)	2 (+++)	0 (+++)
	24 (ND)	38 (ND)	32 (ND)	40 (ND)	34 (ND)	40 (ND)

*Ent. faecium* (40)	6 (+)	6 (+)	6 (+)	8 (+)	8 (+)	6 (+)
	4 (++)	4 (++)	6 (++)	4 (++)	2 (++)	6 (++)
	2 (+++)	0 (+++)	0 (+++)	0 (+++)	2 (+++)	0 (+++)
	28 (ND)	30 (ND)	28 (ND)	28 (ND)	28 (ND)	28 (ND)

*L*. *mesenteroides* (41)	4 (+)	5 (+)	4 (+)	6 (+)	4 (+)	3 (+)
	1 (++)	0 (++)	0 (++)	0 (++)	3 (++)	2 (++)
	0 (+++)	0 (+++)	0 (+++)	0 (+++)	0 (+++)	0 (+++)
	36 (ND)	36 (ND)	37 (ND)	35 (ND)	34 (ND)	36 (ND)

*Ent. italicus* (39)	6 (+)	11 (+)	4 (+)	6 (+)	10 (+)	6 (+)
	3 (++)	0 (++)	5 (++)	0 (++)	3 (++)	4 (++)
	0 (+++)	0 (+++)	0 (+++)	0 (+++)	0 (+++)	0 (+++)
	30 (ND)	28 (ND)	26 (ND)	33 (ND)	26 (ND)	29 (ND)

*L. helveticus* (35)	5 (+)	6 (+)	2 (+)	4 (+)	4 (+)	2 (+)
	0 (++)	3 (++)	2 (++)	3 (++)	1 (++)	3 (++)
	0 (+++)	0 (+++)	3 (+++)	0 (+++)	0 (+++)	0 (+++)
	30 (ND)	26 (ND)	28 (ND)	28 (ND)	30 (ND)	30 (ND)

^*^(+) <1–4 cm, (++) >4–8 cm, (+++) >8–12 cm and (ND) = Not detected. *B*.: *Bacillus*, *E*.: *Escherichia*,* L*.:* Listeria, S.: Salmonella, Staph.: Staphylococcus *and* P.*: *Pseudomonas*.

**Table 4:** Amino acids profile (mg/L) of *Nunu* prepared with various single and combined starter culture(s) of LAB.

Amino acids	Starter cultures										
	Sp	Lf	Lp	Lh	Lm	Lf + Lp	Lf + Lh	Lf + Lm	Lp + Lh	Lp + Lm	Lh + Lm
Lysine^*^	2.53 ± 0.02^h^	4.60 ± 0.06^a^	3.80 ± 0.02^b^	4.65 ± 0.02^a^	1.91 ± 0.01^f^	2.43 ± 0.01^e^	3.85 ± 0.01^b^	2.63 ± 0.02^d^	3.53 ± 0.06^c^	2.65 ± 0.02^d^	2.12 ± 0.01^g^
Argentine	3.15 ± 0.01^a^	6.88 ± 0.09^b^	3.44 ± 0.01^c^	4.32 ± 0.01^d^	1.93 ± 0.01^e^	4.94 ± 0.02^f^	3.51 ± 0.01^g^	ND	3.63 ± 0.01^h^	1.55 ± 0.01^i^	ND
Histidine^*^	2.93 ± 0.06^a^	3.85 ± 0.09^d^	4.11 ± 0.01^e^	3.83 ± 0.03^d^	3.57 ± 0.03^c^	3.00 ± 0.00^b^	2.96 ± 0.01^b^	ND	3.97 ± 0.06^f^	3.10 ± 0.10^b^	ND
Methionine^*^	1.75 ± 0.02^a^	4.58 ± 0.02^e^	3.99 ± 0.01^f^	2.13 ± 0.02^c^	1.88 ± 0.01^b^	4.88 ± 0.01^g^	5.73 ± 0.02^h^	1.88 ± 0.01^b^	6.77 ± 0.01^i^	2.24 ± 0.02^d^	2.24 ± 0.02^d^
Isoleucine^*^	2.99 ± 0.01^a^	5.80 ± 0.11^b^	2.11 ± 0.01^c^	6.51 ± 0.02^e^	3.49 ± 0.01^f^	4.22 ± 0.01^g^	2.86 ± 0.01^a^	2.95 ± 0.00^a^	7.10 ± 0.10^h^	1.16 ± 0.01^i^	2.57 ± 0.02^j^
Tryptophan	3.13 ± 0.01^a^	2.34 ± 0.02^b^	3.42 ± 0.02^c^	3.95 ± 0.01^d^	2.01 ± 0.01^e^	4.86 ± 0.01^f^	2.51 ± 0.01^g^	3.66 ± 0.01^h^	5.27 ± 0.06^i^	ND	ND
Threonine^*^	3.33 ± 0.02^a^	3.21 ± 0.01^b^	2.82 ± 0.01^c^	4.07 ± 0.15^d^	ND	4.53 ± 0.01^e^	2.52 ± 0.01^f^	3.32 ± 0.01^a^	ND	1.46 ± 0.01^g^	ND
Proline	2.63 ± 0.01^a^	2.68 ± 0.02^a^	3.77 ± 0.01^c^	4.51 ± 0.02^d^	0.80 ± 0.01^e^	4.67 ± 0.01^b^	3.44 ± 0.02^f^	4.64 ± 0.01^b^	4.75 ± 0.04^b^	1.90 ± 0.02^g^	2.79 ± 0.02^h^
Glutamine	4.02 ± 0.01^a^	4.22 ± 0.01^g^	2.68 ± 0.00^d^	7.82 ± 0.02^i^	0.98 ± 0.01^b^	3.80 ± 0.01^f^	3.77 ± 0.02^f^	2.68 ± 0.01^d^	5.47 ± 0.12^h^	3.14 ± 0.02^e^	1.97 ± 0.01^c^
Asparagine	3.21 ± 0.01^f^	2.45 ± 0.01^c^	2.55 ± 0.01^d^	4.70 ± 0.01^g^	1.58 ± 0.02^a^	2.95 ± 0.01^e^	3.22 ± 0.01^f^	1.53 ± 0.01^a^	6.41 ± 0.01^h^	2.61 ± 0.01^d^	1.99 ± 0.01^b^
Glutamic acid	3.14 ± 0.01^f^	2.65 ± 0.02^d^	3.24 ± 0.05^f^	4.50 ± 0.01^g^	2.92 ± 0.02^e^	ND	2.45 ± 0.02^c^	2.09 ± 0.01^a^	7.10 ± 0.01^h^	3.18 ± 0.01^f^	2.39 ± 0.02^c^
Valine^*^	2.81 ± 0.02^c^	ND	4.11 ± 0.01^e^	4.21 ± 0.01^f^	4.87 ± 0.02^g^	2.47 ± 0.01^b^	2.46 ± 0.02^b^	2.96 ± 0.01^d^	ND	2.16 ± 0.01^a^	2.95 ± 0.01^d^
Phenylamine^*^	1.18 ± 0.01^a^	3.83 ± 0.02^e^	4.07 ± 0.16^f^	3.62 ± 0.01^d^	ND	4.74 ± 0.01^h^	1.94 ± 0.02^b^	ND	4.45 ± 0.01^g^	3.96 ± 0.01^f^	2.92 ± 0.02^c^
Aspartic acid	1.25 ± 0.01^a^	3.54 ± 0.06^d^	3.71 ± 0.02^e^	4.07 ± 0.01^f^	ND	ND	2.81 ± 0.02^b^	3.27 ± 0.01^c^	4.45 ± 0.01^g^	ND	ND
Serine	ND	ND	2.66 ± 0.02^a^	4.74 ± 0.01^c^	ND	ND	6.33 ± 0.01^b^	3.28 ± 0.01^c^	5.32 ± 0.01^g^	ND	ND
Glycine	2.14 ± 0.02^b^	4.23 ± 0.01^h^	3.24 ± 0.02^e^	3.22 ± 0.01^e^	2.51 ± 0.01^c^	ND	4.07 ± 0.01^g^	3.42 ± 0.01^f^	5.57 ± 0.01^i^	3.12 ± 0.01^d^	1.77 ± 0.02^a^
Tyrosine	1.16 ± 0.01^a^	2.78 ± 0.02^d^	5.34 ± 0.02^h^	3.03 ± 0.01^e^	ND	3.70 ± 0.01^f^	2.71 ± 0.01^d^	4.22 ± 0.00^g^	6.00 ± 0.10^i^	2.16 ± 0.01^c^	1.82 ± 0.02^b^
Alanine	1.44 ± 0.01^a^	3.75 ± 0.05^h^	7.96 ± 0.01^j^	2.63 ± 0.02^e^	2.47 ± 0.01^d^	3.70 ± 0.01^h^	3.10 ± 0.00^g^	2.43 ± 0.01^c^	4.32 ± 0.01^i^	2.77 ± 0.02^c^	2.15 ± 0.02^b^
Cystine	2.10 ± 0.01^b^	2.81 ± 0.01^e^	2.70 ± 0.01^d^	5.60 ± 0.01^g^	ND	ND	6.18 ± 0.02^h^	2.41 ± 0.01^c^	4.51 ± 0.06^f^	2.65 ± 0.02^d^	1.59 ± 0.01^a^
Leucine^*^	1.25 ± 0.01^a^	2.61 ± 0.02^b^	3.92 ± 0.01^c^	5.98 ± 0.01^d^	0.76 ± 0.01^e^	5.77 ± 0.01^f^	4.56 ± 0.01^g^	4.86 ± 0.02^h^	6.21 ± 0.01^i^	1.76 ± 0.01^j^	1.19 ± 0.01^k^

^*^Essential amino acids. Values are means of three experiments; ND: not detected; ±: standard deviation. Values in the same row with different letters differ significantly from each other (*P* < 0.05). Spontaneously fermented (Sp), *L. fermentum* (Lf),* L. plantarum *(Lp),* L. helveticus *(Lh), and* L. mesenteroides* (Lm), *L. fermentum* +* L. plantarum *(Lf + Lp),* L. fermentum* +* L. helveticus* (Lf + Lh), *L. fermentum + L. mesenteroides *(Lf + Lm), *L. plantarum + L. helveticus *(Lp + Lh),* L. plantarum + L. mesenteroides *(Lp + Lm),* L. helveticus* + *L. mesenteroides *(Lh + Lm).

**Table 5:** Mean score of sensory attributes of *Nunu* prepared with various single and combined starter cultures. Evaluation is based on a nine-point hedonic scale.

Starter cultures	Taste	Odour	Colour/appearance	Texture	Overall acceptability
Sp (control)	6.53 ± 0.06^d^	5.00 ± 0.10^a^	6.40 ± 0.10^b^	6.53 ± 0.06^b^	6.45 ± 0.06^a^
Lf	6.07 ± 0.15^b^	6.53 ± 0.06^d^	7.13 ± 0.06^c^	7.27 ± 0.06^c,d^	7.17 ± 0.12^b^
Lp	7.53 ± 0.06^g^	7.90 ± 0.10^f^	8.03 ± 0.15^e^	8.50 ± 0.20^e^	7.20 ± 0.00^b^
Lh	6.40 ± 0.00^c^	8.43 ± 0.15^g^	7.43 ± 0.15^d^	8.20 ± 0.20^e^	8.00 ± 0.20^e^
Lm	5.00 ± 0.17^a^	6.33 ± 0.06^c^	6.53 ± 0.06^b^	8.43 ± 0.15^e^	7.40 ± 0.17^b,d^
Lf + Lp	8.03 ± 0.15^h^	8.50 ± 0.20^g^	7.13 ± 0.06^c^	7.13 ± 0.12^c^	8.43 ± 0.15^f^
Lf + Lh	7.17 ± 0.06^e^	8.43 ± 0.06^g^	8.03 ± 0.06^e^	8.43 ± 0.06^e^	8.17 ± 0.12^e,f^
Lf + Lm	6.20 ± 0.10^b^	6.10 ± 0.17^b^	7.27 ± 0.15^c,d^	7.43 ± 0.15^d^	7.43 ± 0.06^d^
Lp + Lh	7.03 ± 0.16^e^	8.63 ± 0.06^g^	7.87 ± 0.15^e^	8.50 ± 0.20^e^	7.70 ± 0.10^d^
Lp + Lm	7.30 ± 0.00^f^	5.17 ± 0.06^a^	6.57 ± 0.15^b^	8.30 ± 0.10^e^	7.50 ± 0.26^d^
Lh + Lm	7.50 ± 0.20^g^	7.20 ± 0.10^e^	5.53 ± 0.06^a^	6.23 ± 0.15^a^	6.50 ± 0.20^a^

Values are means of two experiments, ±: standard deviation. Values in the same column with different letters differ significantly from each other (*P* < 0.05). Spontaneously fermented (Sp), *L. fermentum* (Lf), *L. plantarum *(Lp),* L. helveticus *(Lh), and* L. mesenteroides* (Lm), *L. fermentum* +* L. plantarum *(Lf + Lp),* L. fermentum* +* L. helveticus* (Lf + Lh), *L. fermentum + L. mesenteroides *(Lf + Lm), *L. plantarum + L. helveticus *(Lp + Lh),* L. plantarum + L. mesenteroides *(Lp + Lm),* L. helveticus + L. mesenteroides *(Lh + Lm).
